# Flying Fox, Vincent van Gogh, 1885

**DOI:** 10.3201/eid0803.030300

**Published:** 2002-03

**Authors:** Paul Arguin

**Affiliations:** Centers for Disease Control and Prevention, Atlanta, Georgia

**Figure:**
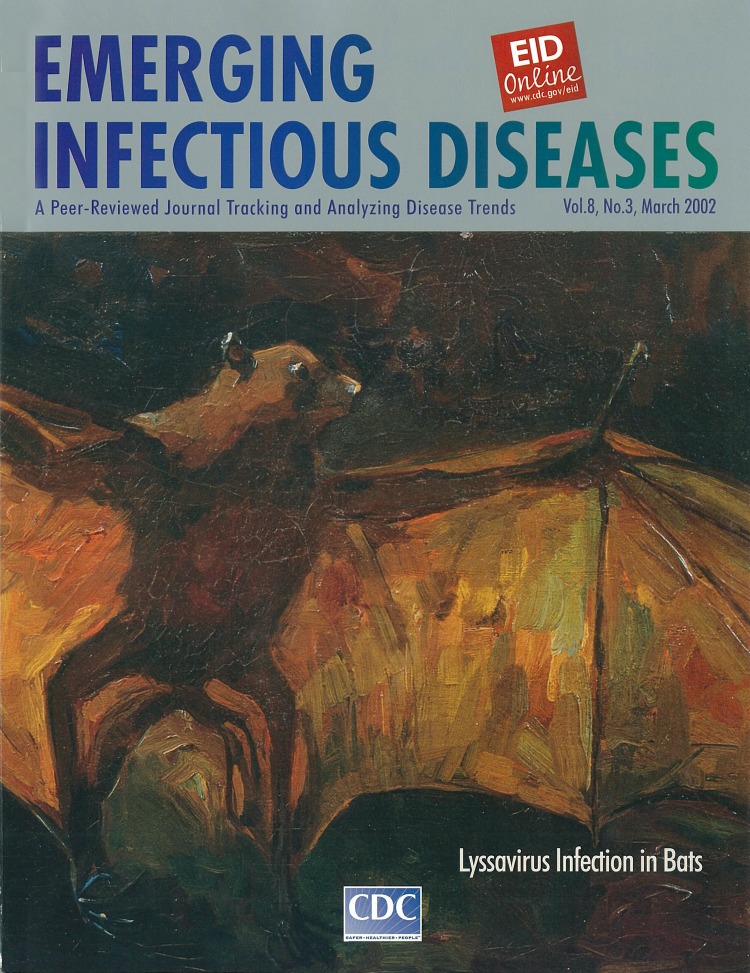
**Flying Fox, Vincent van Gogh, 1885.** (oil on canvas, 41 cm x 79 cm). Courtesy of Van Gogh Museum, Amsterdam (Vincent van Gogh Foundation)

According to the Van Gogh Museum in Amsterdam, the origins of Flying Fox are not well documented. Van Gogh probably saw a flying fox in a museum or private collection in Brabant, Antwerp, or Paris. The dark brown background colors in the painting are similar to those in other works of his Nuenen period. The brighter colors and rough brushstrokes in the wings are more avant garde and suggest techniques used in his later paintings.

Flying foxes like the one that captured van Gogh’s imagination are very large fruit-eating bats (order Chiroptera, suborder Megachiroptera). These mammals are found in tropical and subtropical regions between Africa and the South Pacific, including the Philippines, where there are 70 species of bats. Flying foxes can weigh as much as 1.5 kg and have a wingspan of up to 1.8 m. Occasionally, they are hunted and used as a food source (*1*).

Worldwide, bats are a major predator of night-flying insects and farm pests. Throughout the tropics, they are vital to the survival of the rain forest through their seed dispersal and pollination activities. Studies of bats have contributed to medical advances, including the development of navigational aids for the blind.
